# Solid-to-Solid Manufacturing Processes for High-Performance Li-Ion Solid-State Batteries

**DOI:** 10.3390/polym17131788

**Published:** 2025-06-27

**Authors:** David Orisekeh, Byeong-Min Roh, Xinyi Xiao

**Affiliations:** 1 Department of Mechanical Engineering, University of North Texas, Denton, TX 76205, USA; davidorisekeh@my.unt.edu; 2 School of Industrial and System Engineering, University of Oklahoma, Norman, OK 73019, USA; roh@ou.edu

**Keywords:** solid-state battery, additive manufacturing, polymer, solid electrolyte, separator, electrode, ionic conductivity, mechanical property

## Abstract

Batteries are used as energy storage devices in various equipment. Today, research is focused on solid-state batteries (SSBs), replacing the liquid electrolyte with a solid separator. The solid separators provide electrolyte stability, no leakage, and provide mechanical strength to the battery. Separators are mostly manufactured by either traditional processes or 3D printing technologies. These processes involve making a slurry of plastic, active and conductive material and usually adding a plasticizer when making thin films or filaments for 3D printing. This study investigates the additive manufacturing of solid-state electrolytes (SSEs) by employing fused deposition modeling (FDM) with recyclable, bio-derived polylactic acid (PLA) filaments. Precise control of macro-porosity is achieved by systematically varying key process parameters, including raster orientation, infill percentage, and interlayer adhesion conditions, thereby enabling the formation of tunable, interconnected pore networks within the polymer matrix. Following 3D printing, these engineered porous frameworks are infiltrated with lithium hexafluorophosphate (LiPF_6_), which functions as the active ionic conductor. A tailored thermal sintering protocol is then applied to promote solid-phase fusion of the embedded salt throughout the macro-porous PLA scaffold, resulting in a mechanically robust and ionically conductive composite separator. The electrochemical ionic conductivity and structural integrity of the sintered SSEs are characterized through electrochemical impedance spectroscopy (EIS) and standardized mechanical testing to assess their suitability for integration into advanced solid-state battery architectures. The solid-state separator achieved an average ionic conductivity of 2.529 × 10^−5^ S·cm^−1^. The integrated FDM-sintering process enhances ion exchange at the electrode–electrolyte interface, minimizes material waste, and supports cost-efficient, fully recyclable component fabrication.

## 1. Introduction

With the advancement of next-generation technologies, efficient and renewable energy storage devices have continued to experience a growth in demand, especially for use in artificial intelligence (AI), portable electronic devices, robotics devices, and electric vehicles (EVs) [1]. The future applications of batteries have been projected to increase in industries such as electric flights [2,3]. EV demand has been increasing for the past decade: in 2023, the global EV fleet (car, bus, van, and truck) sales from the different manufacturers were over 14 million and are projected to double by 2035 [4,5,6]. Among the various batteries in use, the applications of lithium-ion batteries (LIBs) in electronic devices have continued to increase since its invention in 1991 [7]. This high demand for LIBs could be attributed to their tremendous improvement in performance, low cost, high specific capacity, and voltage [1,8].

Energy storage devices include electrochemical storage, chemical storage, mechanical storage, and thermal storage [9]. Electrochemical storage includes batteries and superconductors [10], and they provide high-energy density and high-power density, which are used to power electrical devices [11,12,13]. Electrochemical devices convert chemical energy to electrical energy, which could be reversed by passing electron and ion transfer in electrodes through a reduction process [14]. Batteries are made of a combination of electrolytes and electrodes; the electrode is composed of the anode and cathode. The electrons flow into the electrochemical cell through the cathode and flow out through the anode during discharge. The electrolyte is the medium through which ions are transferred between the anode and cathode [15]. Batteries provide high relative energy density but have low power density, which means they take about 2–6 h to recharge [11]. The conventional commercially available LIBs make use of liquid electrolytes, and the liquid electrolytes are chemically unstable [16], creating significant environmental safety risks in the form of fire and explosion [1,17]. These explosions are caused due to the presence of highly flammable and volatile carbonates [18,19]. A promising alternative to the environmental and safety risk linked to liquid LIBs is the use of solid electrolytes in the manufacturing of all-solid-state batteries (SSBs) [16,20,21]. All-solid-state batteries use solid materials as electrolyte, anode, and cathode [20]. According to Randau et al., SSBs with metal lithium negative electrodes have higher energy density compared to LIBs [22]. SSEs are less hazardous than batteries using liquid electrolytes, they are more environmentally friendly with good thermal and chemical stability, non-electrolyte leakages, non-volatile, non-corrosive, non-explosive, have high reliability, have a simple cell structure, have a long shelf life, have high modulus, and ease in manufacturing to various shapes [8,17,22], which makes them ideal for future-generation energy storage technology. The environmental impact of SSBs has been studied by various researchers. It has been found that SSBs have the least global warming potential, and ozone depletion potential, and possess a lesser environmental safety risk compared to LIBs [23,24,25,26,27].

**Figure 1:**
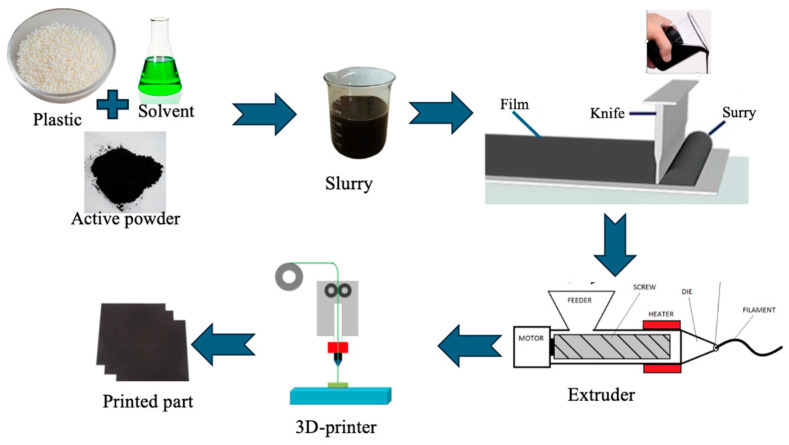
Conventional solid electrolyte manufacturing processes.

Various materials for separators and electrodes have been used for LIBs. The most used active materials for the separator include lithium hexafluorophosphate (LiPF_6_), lithium tetrafluoroborate, and lithium perchlorate. Cathode materials include lithium nickel manganese cobalt oxide, lithium iron phosphate (LFP), lithium manganese iron phosphate, lithium cobalt oxide (LCO), and lithium manganese oxide (LMO). Anode material includes natural graphite powder, lithium titanate, and mesocarbon microbead synthetic graphite [28]. Conductive materials are added to the cathode and/or electrolyte to increase the ionic conductivity; such materials include carbon black, carbon nanotube, and coal tar pitch carbon [29,30,31]. All-solid-state batteries can be manufactured using various manufacturing techniques. Battery components’ manufacturing and assembly processes have a significant effect on the performance [7], therefore, the manufacturing process is of ultimate importance. One better approach is using additive manufacturing [28]. AM, also known as three-dimensional (3D) printing, is the layer-by-layer addition of material by a 3D printer until the desired part or shape is achieved. AM technology is preferred in the manufacturing of energy storage devices when a complex or intricate shape is desired; it produces a better layer interface and reduces printing material waste [32,33,34]. Although many studies have been performed on solid-state-LIB manufacturing processes, especially using 3D-ink-writing printing technology, which involves making a slurry of the separator materials, these processes involve material wastage from the slurry and are more expensive and with many steps, as seen in Figure 1.

Various researchers have used different materials and processes to manufacture solid-state electrodes and electrolytes. Orisekeh et al. (2022) [16] fabricated a solid-state electrolyte using a solid polymer blend mixture of polyvinylpyrrolidone/polyvinyl alcohol (PVP/PVA) and lithium perchlorate salt (LiClO_4_). The results show a Young’s modulus valve of 6.87 GPa, a hardness value of 1.3 GPa, and tensile strength of 4.3 MPa. Sun et al. (2013) [35] fabricated a 3D interdigitated micro-battery architecture (3D-IMA) made of lithium iron phosphate, LiFePO_4_ (LFP), and lithium titanate, Li_4_Ti_5_O_12_ (LTO), as cathode and anode materials, respectively. This technique is used to print parts as low as 1 μm using prepared ink through a printer deposition nozzle of 30 μm. The cathode and anode inks were prepared by suspending LFP and LTO nanoparticles in deionized water, ethylene glycol, and glycerol solution. The electrical resistivity results of the cathode and anode were 2.3 × 10^3^ Ω·cm and 2.1 × 10^5^ Ω·cm, respectively.

Fused filament fabrication (FFF) is one 3D printing technique used by many researchers [36]. Foster et al. (2017) [37] used graphene-based polylactic acid filament to 3D print disc-like electrodes for solid-state batteries. The architecture of the anode is designed as a freestanding solid-state superconductor that does not require a current collector at the negative end of the battery, thereby reducing the cost of production compared to the conventional Li-ion-based battery setup. This is achieved by using a RepRap FDM 3D printer to fabricate a graphene-based lithium-ion anode with the ability to electrochemically produce hydrogen via the hydrogen evolution reaction (HER). This is an alternative replacement for platinum-based electrodes. The electrochemical characterization gave a value of 1.0 × 10^−3^ Scm^−1^ for the graphene/PLA sample. A similar study was carried out by Fu et al. (2016) [38], who used graphene oxide-based electrodes and electrolyte inks for 3D printed lithium-ion batteries. Graphene oxide has shown a good printing capacity and good viscoelastic properties, and it possesses good electrical conductivity after thermal annealing. The electrodes were freeze-dried and thermally annealed to remove the electrode’s (water) solvent. The sample showed initial charge and discharge capacities of 168 and 164 mAh g^−1^, respectively, which is close to the theoretical value of 170 mAh g^−1^. According to Wei et al. (2017) [39], the direct ink writing 3D printing technique for printing energy storage devices provides higher power and energy density because it increases the surface area of electrodes compared to traditional techniques.

**Figure 2:**
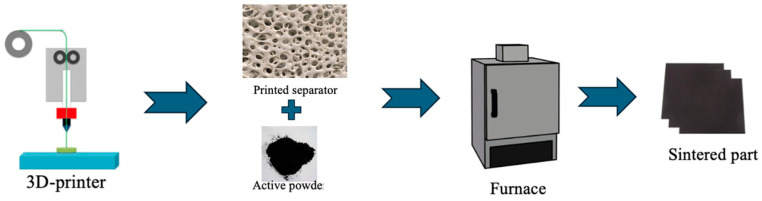
Battery separator proposed by this study.

Conventional slurry-based coating, wet impregnation, and solution-casting methods introduce residual solvents or water into the electrolyte structure, which can lead to uncontrolled interfacial reactions, incomplete solvent removal, and the formation of undesired by-products such as lithium hydroxides or carbonates, ultimately degrading the ionic conductivity and long-term stability of the solid electrolyte. Moreover, exposure to moisture during processing can compromise the stoichiometry of highly reactive lithium-based ceramic or glassy electrolytes, such as lithium lanthanum zirconium oxide (LLZO) or sulfide-based materials, resulting in diminished electrochemical performance and increased interfacial resistance when paired with lithium metal anodes. Therefore, a fully dry, solvent-free manufacturing strategy—such as FDM—provides precise control over material purity, microstructural homogeneity, and interfacial compatibility [40,41,42,43,44]. This study seeks to provide a novel manufacturing process for the fabrication of solid electrolytes for solid-state LIBs with no liquid or aqueous material involved throughout the process, as shown in Figure 2.

This approach eliminates the need for subsequent high-vacuum drying or high-temperature solvent removal steps, reduces processing complexity, and enhances scalability and environmental sustainability, thereby paving the way for safer, more reliable, and cost-effective production of solid electrolytes for next-generation all-solid-state LIBs.

## 2. Materials and Methods

### 2.1. Materials

PLA filaments of diameter 1.75 mm and density 1.25 g/cm^3^ were purchased from Henan Creatbot Technology Ltd., Zhengzhou, China. Lithium Hexasfluorophosphate (LiPF_6_) powder with a density of 1.50 g/cm^3^, and molecular weight of 151.91 g/mol was purchased from MSE Supplies, Tucson, AZ, USA, and Al_2_O_3_ powder of density 3.987 g/cm^3^, a molecular weight of 101.96 g/mol, and average particle size of 1–2 μm was purchased from MSE Supplies, AZ, USA. Commercial cathode, lithium iron phosphate (LFP), and anode, graphite, coin cell case (20d × 1.6 mm), stainless-steel spacer (15.8d × 1.0 mm), stainless-steel wave spring, and coin cell holder were purchased from MTI Corporation, Richmond, CA, USA.

### 2.2. Separator Design

**Figure 3:**
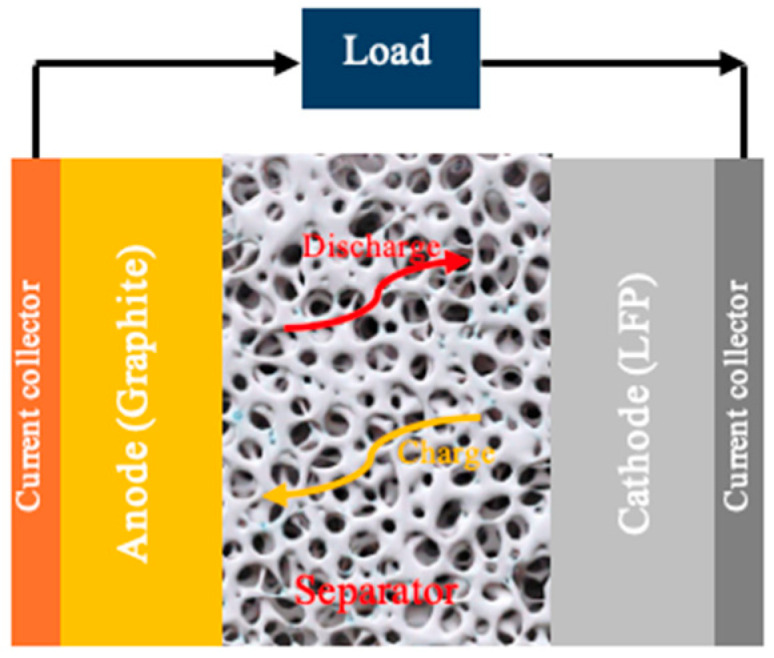
Schematic of a solid-state battery.

Polymers are mostly used as battery separators due to their suitable properties, such as thermal, mechanical, and electrochemical properties [45]. A separator is crucial to the overall battery performance, not just as an electrical insulator between the cathode and anode but as a medium for the movement of ions between the electrodes both during charging as well as during discharging (Figure 3) [45]. Performance is determined by the separator’s porosity; pore size; chemical, thermal, and mechanical properties; and electrolyte absorption capacity [46,47,48]. In this study, the separator was printed using PLA, with an infill density of 20% and an infill line distance of 0.8. This was done to increase porosity and pore size. Previous studies have reported that effective solid-state battery separators typically exhibit porosity levels ranging from 20% to 80%, with pore sizes below 2 μm, in order to facilitate high ionic conductivity while maintaining mechanical integrity [49,50]. These structural parameters are critical for enabling efficient Li^+^ ion transport while preventing dendrite growth and maintaining interfacial contact with electrodes. In this study, the separator was intentionally engineered with tailored porosity by employing a 20% infill density using a gyroid infill pattern during the FDM printing process. The gyroid architecture, a triply periodic minimal surface (TPMS) structure, was selected due to its continuous and interconnected pore network, which optimizes both ion diffusion pathways and structural robustness.

Following fabrication, the porous samples were infiltrated with lithium hexafluorophosphate (LiPF_6_) powder. During the subsequent sintering process, the electrolyte particles were thermally anchored within the polymer matrix, becoming physically trapped in the internal voids formed by the gyroid structure. This entrapment ensures stable ionic domains within the separator and provides continuous ion-conduction channels throughout the bulk of the material. The interconnected pore geometry, combined with the uniform distribution of LiPF_6_, facilitates rapid and efficient Li^+^ transport during battery charge and discharge cycles, contributing to enhanced ionic conductivity and overall electrochemical performance of the solid-state battery system. This design strategy demonstrates the potential for additive manufacturing to precisely tune porosity and phase distribution in multifunctional energy materials.

**Figure 4:**
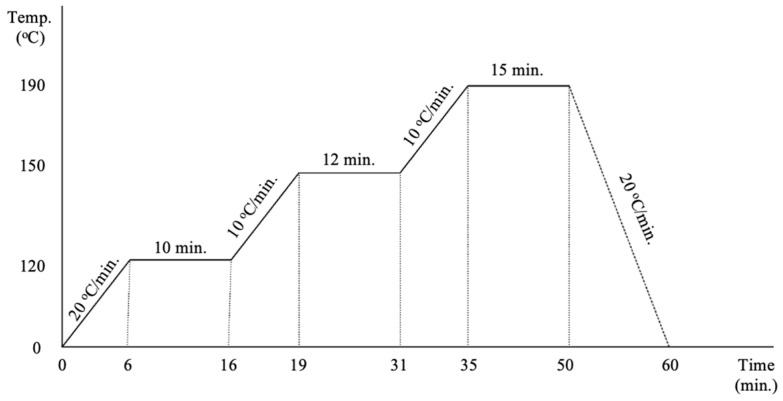
The separator sintering profile.

The separator was designed with dimensions D(16) × T(0.6) mm^3^. The samples were 3D printed using CreatBot PEEK-300, (CreatBot, Zhengzhou, China). Table 1 shows the printing parameters. The porous PLA-based samples fabricated via FDM, along with lithium hexafluorophosphate (LiPF_6_) powder, were subjected to a controlled sintering process using a high-precision programmable furnace (PHOENIX-9C, Anyang Yingpai Dental Material Co., Ltd., Anyang, China). The sintering profile, detailed in Figure 4, involved a multi-stage heating protocol optimized to ensure gradual polymer degradation, salt infiltration, and solid-state fusion without thermal decomposition of LiPF_6_. The profile includes a ramp-up stage to approximately 150 °C to promote polymer softening and salt absorption, followed by a plateau stage around 190–200 °C to enable sintering of the LiPF_6_ within the porous matrix while preserving its ionic activity. The resulting sintered separators maintain structural integrity with a visibly fused salt phase distributed across the pore network. LiPF6, in the presence of moisture, can decompose, producing hydrofluoric acid (HF). HF is generally corrosive, which can cause damage to battery components, like current collectors. Al_2_O_3_ (aluminum oxide) is usually added to the separator’s surface to limit the effect of HF. A secondary sintering step was performed by co-sintering the separator with a thin coating of Al_2_O_3_ powder. The Al_2_O_3_ was uniformly deposited onto the surface of the sintered sample prior to reheating under a modified thermal profile that prevents PLA degradation while promoting ceramic bonding. This surface modification serves two purposes: (1) it improves the separator’s wettability and adsorption capacity for liquid or gel-phase electrolytes, and (2) it passivates the surface against thermal and chemical degradation during extended cycling. The literature reports [51,52] indicate that Al_2_O_3_-modified separators exhibit enhanced interfacial compatibility with electrodes and reduced interfacial resistance, which is critical for long-term cyclability and charge transport stability in solid-state battery applications.

### 2.3. Sample Composition

The mass of the printed pristine PLA sample was weighed before sintering, the density was known, and the volume was calculated using Equation (1). The sintered sample was weighed, and the mass of PLA was subtracted to obtain the mass of LiPF_6_ in the sample, and from the density, the volume of LiPF_6_ was calculated (Table 2).

(1) $$v=\frac{m}{\rho }$$

where *v* is volume, *m* is mass, and ρ is density.

### 2.4. Cell Assembling

The coin cells were assembled using a digital pressure-controlled electric crimper supplied by MTI Corporation, Richmond, CA, USA. This is used to apply adequate pressure to ensure the proper surface-to-surface interaction between the cathode and separator and anode and separator. The coin cells were inserted into the coin cell holder for electrochemical impedance spectroscopy (EIS) analysis. A circular copper foil with a diameter of 15.0 mm, uniformly coated on one side with an active graphite layer (typical areal loading of ~2–3 mg/cm^2^), was employed as the anode and carefully positioned on the positive casing side (stainless steel) of the coin cell assembly. A macro-porous separator, which is produced using the abovementioned manufacturing strategy, was then placed directly atop the anode to electrically isolate it from the cathode while allowing efficient lithium-ion transport. Subsequently, a 15.0 mm diameter aluminum foil coated on one side with lithium iron phosphate (LiFePO_4_) cathode material (with an areal loading typically ranging from 3 to 5 mg/cm^2^) was aligned concentrically above the separator. A stainless-steel coil spring and a spacer were inserted on top of the cathode to ensure uniform compressive force was applied across the electrode stack upon crimping, promoting intimate interfacial contact and minimizing interlayer resistance. In this study, the anode disc was fabricated with a slightly smaller active area than the cathode to prevent the risk of edge short-circuiting due to misalignment or deformation of the electrode layers during assembly or cycling. This configuration ensures robust cell integrity, consistent electrochemical performance, and safe operation under standard charge–discharge conditions.

## 3. Results

### 3.1. Electrochemical Impedance Spectroscopy (EIS) Analysis

**Figure 5:**
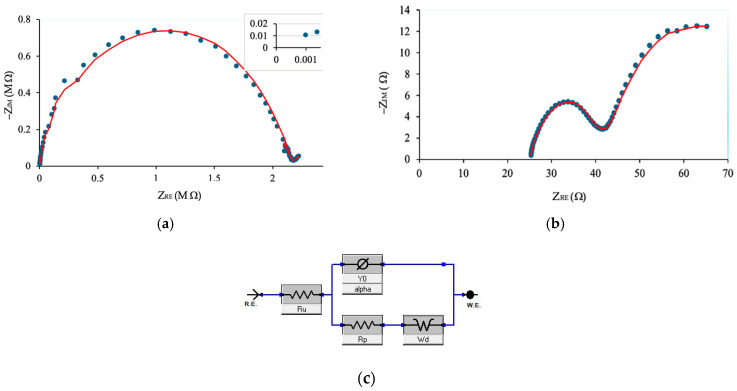
Nyquist plot obtained at room temperature: (**a**) solid electrolyte, (**b**) liquid electrolyte, and (**c**) equivalent circuit model.

The ionic conductivity of the polymer/LiPF_6_-based solid-state separator was characterized using electrochemical impedance spectroscopy (EIS) on a Gamry Interface 101E potentiostat/galvanostat (Gamry Instruments, Warminster, PA, USA). The test samples were assembled into standard CR2032 coin cell holders with stainless-steel blocking electrodes, ensuring consistent contact and geometry for impedance measurement. Electrical connections were established between the coin cell terminals and the potentiostat to enable impedance monitoring across the separator material. The EIS measurements were conducted by applying a small-amplitude (10 mV) sinusoidal perturbation superimposed on a zero or low DC bias potential, sweeping logarithmically from a high initial frequency of 1 MHz down to 0.1 Hz [30]. This wide frequency range captures both bulk and interfacial processes within the separator. Impedance data were recorded and analyzed using Gamry Echem Analyst software, version 7.10. The resulting Nyquist plots, shown in Figure 5a,b for the solid-state and liquid electrolyte configurations, respectively, display the complex impedance behavior of each system. For both cases, the bulk resistance R_b_ was extracted from the high-frequency intercept of the semicircular region with the real axis (Z′). The comparison between the Nyquist plots of solid and liquid systems underscores the trade-off between mechanical robustness and ionic conductivity, providing insight into optimization pathways for additive-manufactured solid-state battery components. Figure 5c illustrates the equivalent circuit model of the constant phase element (CPE), which follows the Randles equivalent circuit. The solution resistance (R_u_) represents the electrolyte resistance, reflecting the opposition to ion movement within the separator. The polarization resistance (R_p_) corresponds to the resistance encountered during ion transfer at the electrode–electrolyte interface; the lower the value of R_p_, the faster the ion movement across the interface, and the higher the ionic conductivity of the model. The CPE incorporates two parameters: the constant phase element (YO), which replaces a pure capacitor, and alpha (α), indicating the deviation from ideal capacitive behavior. The Warburg element (W_d_) models the impedance associated with diffusion processes.

### 3.2. Mechanical Property Analysis

**Figure 6:**
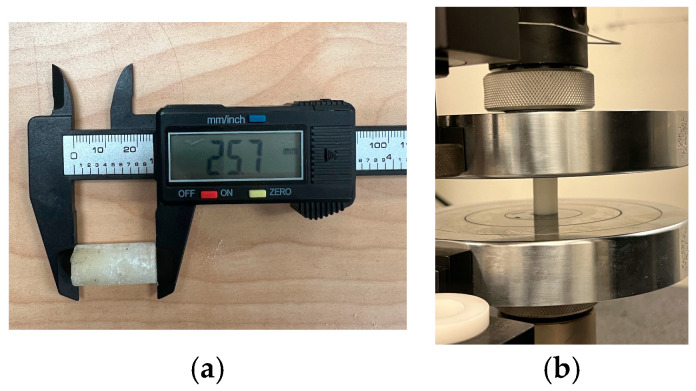
Compression test: (**a**) sample dimension and (**b**) sample testing.

The compressive properties of the polymer/LiPF_6_ were investigated using the universal testing machine INSTRON 5982, Illinois Tool Works Inc., Norwood, MA, USA, as shown in Figure 6b. The samples were prepared according to American Society for Testing and Materials (ASTM) standards D695 [53], with a test speed of 1.3 mm/min and preload of 500 N. Figure 6a shows the sample’s dimension.

Three distinct sample types were fabricated to investigate the influence of porosity and electrolyte integration on the mechanical behavior of the separator: (1) a fully dense control sample consisting of neat PLA printed at 100% infill density; (2) a porous PLA sample printed at 20% infill density using a gyroid structure to replicate the separator geometry without electrolyte integration; and (3) a composite separator fabricated with 20% infill PLA and subsequently sintered with lithium hexafluorophosphate (LiPF_6_) powder infiltrated into its porous network. These samples were prepared under identical printing conditions using a nozzle temperature of 200 °C and a bed temperature of 60 °C to ensure uniform layer adhesion and geometric consistency.

**Figure 7:**
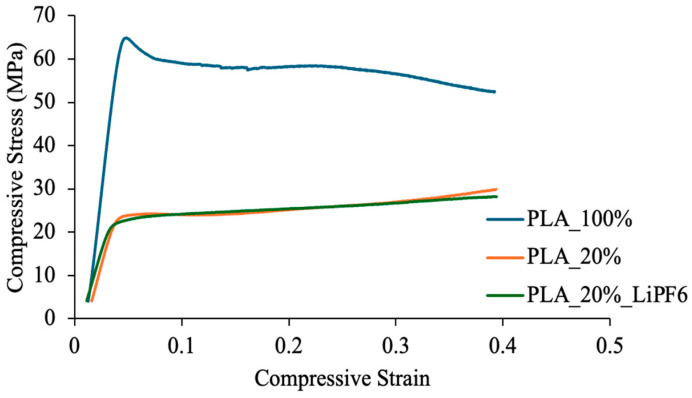
Compressive stress versus compressive strain curve of the samples.

Uniaxial compression tests were performed on all three sample types using the universal testing machine INSTRON 5982, by Illinois Tool Works Inc., Norwood, MA, USA at a constant crosshead speed of 1 mm/min until failure or significant deformation. The specimens were dimensionally standardized with identical diameter-to-height ratios to ensure valid mechanical comparisons. As illustrated in Figure 7 the compression stress–strain plots highlight distinct mechanical responses corresponding to each sample type. The 100% infill PLA exhibited the highest compressive strength and modulus, which was attributed to its solid, defect-free internal structure. The 20% infill PLA displayed significantly reduced mechanical performance due to its engineered porosity and reduced load-bearing cross-sectional area. The sintered PLA/LiPF_6_ composite showed further reductions in compressive strength, primarily due to the presence of sintered salt phases occupying the pore network, which compromise structural integrity while enabling ionic functionality. These results underscore the mechanical trade-offs introduced by porosity and electrolyte integration in solid-state battery separator design. Future optimization will require balancing mechanical stability with ionic transport performance, possibly through the inclusion of reinforcement phases or advanced infill patterns.

## 4. Discussion

### 4.1. Ionic Conductivity

**Figure 8:**
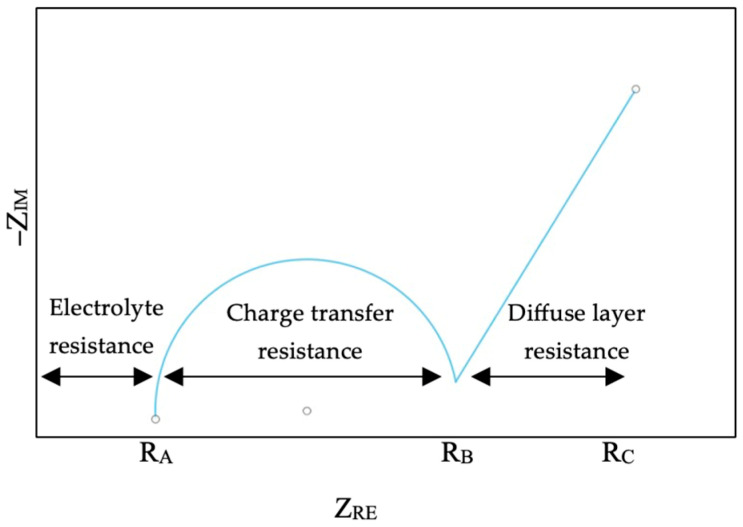
Schematic of a Nyquist plot.

Electrochemical impedance spectroscopy (EIS) is typically conducted at higher frequencies to assess the internal resistance of a battery. In EIS, a small alternating current (AC) signal is applied across different frequencies to a battery and measures the resulting impedance. The impedance related to electron transfer at the electrode interface and through the solid electrolyte interphase (SEI) layer is also crucial for optimizing battery performance. This method is non-invasive and does not require disassembling the battery, making it safe to monitor internal battery behavior. A Nyquist plot is a graphical representation that shows impedance values at various frequency ranges during the test, allowing for the assessment of different phenomena in various parts of the battery. At high frequencies, impedance is primarily influenced by the migration of lithium ions in the electrolyte. At low frequencies, usually below 1 Hz, lithium-ion diffusion occurs within the electrode, and at intermediate frequencies, lithium-ion transfer reactions are observed [54,55,56]. The battery’s internal resistance can generally be divided into three main categories: electrolyte resistance, reaction resistance, and diffusion resistance (Figure 8).

From the Nyquist plot, the value of the bulk resistance at the higher frequency can be determined and used to find the ionic conductivity of the battery since the area and thickness are known from using Equation (2).

(2) $$\sigma =\frac{1}{R_{b}}\frac{t}{A}$$

where σ is the ionic conductivity, *R_b_* is the bulk resistance of the separator, *A* is the contact area, and *t* represents the thickness. Using Equation (2), the ionic conductivity of the solid-state electrolyte separator was calculated to be 2.529 × 10^−5^ S·cm^−1^ at room temperature. The relative lower conductivity in the solid-state system compared to the liquid state is primarily attributed to insufficient interfacial contact between the porous separator and the electrode surfaces. Additionally, the inherent tortuosity and confined microstructure within the sintered PLA-LiPF_6_ composite may contribute to restricted Li^+^ mobility compared to the more homogeneous ion-conduction channels in liquid systems. Addressing interfacial impedance through surface engineering or interface modifiers remains a key focus for improving performance in future iterations. It is generally accepted that to meet the threshold for practical battery performance, solid electrolytes should achieve an ionic conductivity greater than 1 × 10^−4^ S·cm^−1^ at ambient temperature. Table 3 presents a comparison of ionic conductivity values reported in the literature for various polymer/LiPF_6_-based solid electrolytes, including poly(ethylene glycol) (PEG), lignocellulose (LC), poly(ethylene oxide) (PEO), and poly(poly(ethylene glycol) methacrylate) (pPEGMA) matrices. These polymer systems typically exhibit room-temperature ionic conductivities in the range of 10^−5^ to 10^−3^ S·cm^−1^, depending on polymer chain mobility, crystallinity, and the degree of LiPF_6_ dissociation within the matrix. For instance, PEG- and PEO-based electrolytes benefit from segmental motion that facilitates Li^+^ transport through coordinated ether oxygen sites, while LC-based systems leverage natural porosity and hydroxyl-rich networks for salt dispersion. In this study, achieving this benchmark is not sufficient alone; the long-term cyclability and structural stability of the battery are strongly governed by the mechanical integrity of the electrolyte. Materials with low modulus or poor compressive strength may suffer from electrode delamination, cracking, or loss of interfacial contact during repeated charge–discharge cycles. Thus, optimizing both ionic transport properties and mechanical robustness is essential for developing reliable solid-state battery systems.

### 4.2. Mechanical Property

One of the importance functions of the separator is to prevent physical contact between the cathode and anode as this will result in short circuits, which cause the battery to drain very quickly, short out the battery, and could cause a sudden burst of heat, which could lead to a fire. With the rapid growth of the usage of electric vehicles (EVs) due to the progress in battery energy density, road crashes involving EVs could involve intrusion into the battery pack, which will result in fire and explosion. It is, therefore, important to study the mechanical properties of separators. Researchers have studied various mechanical properties of batteries [57,58,59,60]. One very important mechanical property is the compression strength. Figure 7 shows the stress versus strain curve of the separator under compression during loading. From Table 4, neat PLA with 100% infill density shows a compression strength of 61.7 MPa [61], 23.5 MPa for the 20% infill density of neat PLA, and 22.1 MPa for the sintered sample. This result demonstrates that the percentage of infill density affects the compression strength. The sintered sample shows the smallest compression strength as the salt weakens the molecular bond of the polymer due to moisture absorption and sample porosity [58]. In Table 4, a compression modulus of 2.14 GPA for neat PLA with 100% infill density can be seen; a similar value was reported in the literature [53,61]. Sintered PLA/LiPF_6_ shows the smallest modulus of 0.8 GPa, and 0.9 GPa was reported for the 20% infill density of neat PLA (Table 4). The battery separators pose a challenge for compression testing due to the sample’s thickness and porosity, and due to the viscoelasticity of polymer materials. A sample with small thickness and large porosity will facilitate faster ion transport across the separator. Therefore, it is necessary for separator design.

## 5. Conclusions

To develop environmentally friendly, sustainable, and high-energy performance batteries, solid electrolytes have become a new prospect for improving the challenges posed by liquid electrolytes. The solid electrolyte has the advantages of better thermal and mechanical stability, minimal leakage, long cycle life, and low cost. In this study, the solid electrolyte was manufactured as a separator in a battery to replace the conventional liquid electrolyte using PLA and LiPF_6_. The porous 3D separator was sintered in an electric oven to entrap the lithium salt that had infiltrated. Coin cell batteries were assembled, and an EIS test and analysis were carried out to determine the ionic conductivity of the battery. This showed an ionic conductivity in the 10^−5^ S·cm^−1^ range at room temperature, which is a promising result for future research and application. The mechanical properties of the separator were characterized, and the sintered PLA/LiPF_6_ samples showed a decrease in compression properties against the neat PLA. This decrease in a mechanical property has been reported in the literature [48], especially with an increase in lithium salt concentration; therefore, there must be a balance between ionic conductivity and mechanical properties.

The authors are working toward maximizing ionic conductivity in solid-state electrolyte separators in a future work, which aims to design a higher degree of controlled porosity within the polymer scaffold to provide an increased accessible surface area and interconnected pathways for lithium-ion transport. This will be achieved by optimizing key process parameters such as infill density, raster orientation, layer height, and interlayer bonding conditions during AM, thereby tailoring the macro- and meso-porous network within the separator. Additionally, the pore architecture will be engineered to facilitate a uniform infiltration and entrapment of a higher concentration of lithium salt during the post-printing impregnation and subsequent thermal sintering steps. To counteract the trade-off between the bulk ionic resistance and the separator’s mechanical integrity, the polymer matrix—such as PLA or polyvinylidene fluoride (PVDF)—can be reinforced with uniformly dispersed nano-fillers, including ceramic nanoparticles (e.g., Al_2_O_3_, SiO_2_, and TiO_2_) or functionalized nanoclay. These nano-reinforcements serve as mechanical crosslinking agents, enhancing the modulus, fracture toughness, and dimensional stability of the separator while simultaneously providing additional Lewis acid–base interaction sites that may assist in regulating lithium-ion dissociation and transport. This integrated approach can balance a high ionic conductivity with robust mechanical performance, advancing the development of durable, high-efficiency separators for next-generation all-solid-state lithium-ion batteries.

## Figures and Tables

**Table 1:** Separator 3D printing parameters.

Parameter	Value
Printer nozzle diameter	0.4 mm
Printer nozzle temperature	210 °C
Printer bed temperature	45 °C
Printing speed	40%
Infill line distance	0.8 mm
Infill density	20%
Infill pattern	Gyroid
Infill line direction	[0, 90]

**Table 2:** Average separator composition.

	PLA (Vol. %)	LiPF_6_ (Vol. %)
Sample	81	19

**Table 3:** Ionic conductivity comparison from the literature.

	Ionic Conductivity (S·cm^−1^)	Reference
PLA/LiPF_6_	2.529 × 10^−5^	This study
pPEGMA/LiPF_6_	2.07 × 10^−5^	D.P. Nava et al. [30]
PEG/LC/LiPF_6_	3.22 × 10^−4^	C.M. Costa et al. [52]
PEO/LiPF_6_	10^−5^	J. Nunes-Pereira et al. [45]

**Table 4:** Compression properties.

Samples	Compression Strength (MPa)	Compression Modulus (GPa)
PLA 100% infill	61.7	2.14
PLA 20% infill	23.5	0.9
PLA 100% infill/LiPF_6_	22.1	0.8

## Data Availability

Data will be available if requested.

## Abbreviations

The following abbreviations are used in this manuscript:

SSB	Solid-state battery
AM	Additive manufacturing
PLA	Polylactic acid
SE	Solid electrolyte
FFF	Fused filament fabrication
LiPF_6_	Lithium hexafluorophosphate
EIS	Electrochemical impedance spectroscopy
I-C	Ionic conductivity
